# How are psychodynamic conflicts associated with personality functioning? A network analysis

**DOI:** 10.3389/fpsyg.2023.1152150

**Published:** 2023-04-19

**Authors:** Larissa Vierl, Charlotte Von Bremen, York Hagmayer, Cord Benecke, Christian Sell

**Affiliations:** ^1^Department of Psychology, University of Kassel, Kassel, Germany; ^2^Akademie für Psychoanalyse und Psychotherapie München e.V., Munich, Germany; ^3^Georg-Elias-Mueller Institute of Psychology, University of Göettingen, Göttingen, Germany; ^4^International Psychoanalytic University Berlin, Berlin, Germany

**Keywords:** network analysis, Operationalized Psychodynamic Diagnosis (OPD-2), personality functioning, level of structural integration, conflicts

## Abstract

Personality functioning and psychodynamic conflicts are central constructs in psychoanalytic theories of psychopathology as well as in many psychodynamic treatment models. Although there has been a longstanding conceptual discussion on how they relate to each other, empirical evidence on this question is still scarce. In this study, we explore the associations between psychodynamic conflicts and levels of structural integration (which can be used synonymously with personality functioning) by means of a partial correlation network analysis in a sample of *N* = 220 outpatients interviewed and rated according to Operationalized Psychodynamic Diagnosis (OPD-2). We examined network centrality, bridge centrality, clustering, and network stability. The network analysis resulted in separate clusters for levels of structural integration and conflicts, supporting the assumption of distinct psychodynamic constructs. The greatest association between the two clusters was found between the individuation vs. dependency conflict (C1) and the structural capacity to attach to internal objects. In general, C1 showed significantly greater connections with structural dimensions compared to the other five OPD conflicts included. C1 was also more central in the network compared to most other conflicts, whereas the structural dimensions did not differ in centrality. All structural dimensions were found to be strongly interconnected. C1 showed exclusively negative edges to the other conflicts, suggesting that a profound C1 decreases the probability of other psychodynamic conflicts. We discuss clinical as well as conceptual implications of our findings for psychodynamic diagnosis and treatment.

## 1. Introduction

The current revisions of the official diagnostic classification systems feature a new approach for the definition and diagnosis of personality disorders (PDs). In both the Alternative Model for Personality Disorders (AMPD) in the fifth edition of the Diagnostic and Statistical Manual of Mental Disorders (DSM-5; American Psychiatric Association [APA], 2013) and the PD section in the eleventh edition of the International Classification of Diseases (ICD-11; World Health Organization [WHO], 2019) the diagnosis of PDs has shifted toward a dimensional approach. The severity of the PD is now described along a continuum of personality functioning, where impairments in self-functioning and interpersonal functioning on dimensions such as identity, self-direction, empathy, and intimacy are at the core of personality pathology. Maladaptive personality traits can be used to further describe the personality pathology (e.g., negative affectivity, detachment, antagonism, disinhibition, and psychoticism in the AMPD). The result is a hybrid-dimensional-categorical model, where all specific PDs can now be depicted as a combination of a certain impairment in personality functioning and certain maladaptive personality traits.

The revised conception of PDs is inspired by long-established psychoanalytic theories. In particular, the dimensional construct of personality functioning is empirically and conceptually related to contemporary psychodynamic concepts of personality functioning (Clarkin et al., 2020; Blüml and Doering, 2021; Hörz-Sagstetter et al., 2021), such as Kernberg’s (1984) model of personality organization, Fonagy et al.’s (1993) mentalization-based approach, or the levels of structural integration axis of Operationalized Psychodynamic Diagnosis (OPD; OPD Task Force, 2001, 2008, 2023). For more information on the similarities and differences of the psychodynamic theories please see Sell and Benecke (2022). Furthermore, combining a dimensional with a categorical conception of the psyche is also familiar from the psychoanalytic tradition: the OPD, for example, includes both a dimensional conceptualization of personality functioning and an assessment of psychodynamic conflicts (i.e., conflicting inner motivational themes) that can be used similarly to personality traits to further describe and understand a person. However, compared to the AMPD or ICD-11 the OPD is not limited to the conceptualization of PDs but is in principle applicable to everyone, including individuals without any kind of mental disorder.

Concerning the AMPD, in the last decade there has been an ongoing and controversial debate on the inter-relationship between personality functioning and personality traits. Several studies have shown a substantial cross-sectional and conceptual overlap between the two constructs (e.g., Zimmermann et al., 2015; Bach and Hutsebaut, 2018; Hopwood et al., 2018; Widiger and Hines, 2022). Further, incremental validity of personality functioning compared to personality traits alone has often been found to be low or even absent (for an overview see Zimmermann et al., 2019). Some scholars conclude that the separation of the two constructs is redundant and uneconomic. The scholars therefore question the utility of personality functioning and advocate for its abolishment (e.g., Sleep et al., 2019; Sleep and Lynam, 2022; Widiger and Hines, 2022). Yet, Wright and Ringwald (2022) argue that it is reasonable that both constructs are highly correlated, as they both measure personality dysfunction. Further, a study by Sexton et al. (2019) contradicts the statement that personality functioning and traits are redundant concepts, as both were found to interact in a rich and meaningful way. The authors have advised not to collapse the concepts, since both are seen as important for case formulation. Similarly, Kernberg (2016) has warned against a reductionism to personality traits alone. He argues that personality traits are influenced by personality functioning and, thus, that a reductionism would neglect the “complexity of the internal psychological organization of behavior” (Kernberg, 2016, p. 2).

Even though the debate is still going on, it has become clear that understanding the inter-relationship between the different aspects of a hybrid-dimensional-categorical model in diagnostics is important for the system itself as well as for treatment planning. Zimmermann et al. (2015) argue that diagnostic systems should be parsimonious and keep redundancy at a minimum. If two constructs are too similar it would be redundant to use both as they would assess the same phenomenon twice. Also for case formulation and treatment planning it makes a difference if personality functioning and personality traits are considered to be distinct or highly inter-related. Bach and Tracy (2022) stress that from a clinical perspective it is helpful to distinguish personality functioning and personality traits. Clinically, personality functioning can be used for long-term prognosis and optimal treatment intensity, while the personality traits capture several clinically relevant features that can inform the focus and style of the treatment (Bach and Simonsen, 2021).

In the present study we investigate the inter-relationship between personality functioning and psychodynamic conflicts according to the OPD. As mentioned above knowledge of their relationship is not only relevant for (psychodynamic) treatment but is also relevant for the OPD as a diagnostic system. As the OPD has some similarities to the DSM-5 AMPD model, our findings may also contribute to the current debate. We shall commence by introducing the OPD in more detail.

### 1.1. Operationalized Psychodynamic Diagnostics (OPD)

The OPD system was developed as a multiaxial diagnostic and classification system based on psychodynamic principles (OPD Task Force, 2001, 2008, 2023). In its second revision (OPD-2; OPD Task Force, 2008), it encompasses five independent axes: (I) Experience of illness and prerequisites for treatment, (II) interpersonal relations, (III) conflicts, (IV) level of structural integration, and (V) mental and psychosomatic disorders according to the ICD or DSM. The current paper focuses solely on the axes “conflicts” and “level of structural integration.”

#### 1.1.1. Psychodynamic conflicts

Psychodynamic conflicts are understood as time-persistent, mostly unconscious inner motivational themes that shape the person’s experiences and behavior across several areas of life. A conflict initially arises when contrasting demands or motives confront each other within an individual. Their roots often lie in re-occurring experiences, such as conflictual interactions with or specific demands from significant others during the formative years of childhood. These early behavioral patterns are thought to re-emerge in later life, influencing behavior and perception (Benecke et al., 2018). The OPD-2 conflict axis describes seven intra-psychic conflicts: individuation vs. dependency (C1), submission vs. control (C2), need for care vs. autarky (C3), self-worth conflict (C4), guilt conflict (C5), oedipal conflict (C6), and identity conflict (C7). For each of these conflicts, an active and a passive mode of coping is formulated, which describe contrasting ways of dealing with the respective conflict. A short description of the conflicts is given in Table 1. Previous studies have shown that the conflicts C1–C4 are very frequent in clinical populations, while the identity conflict (C7) is only sparsely diagnosed (Pieh et al., 2009; Kaufhold et al., 2017; Schneider and Heuft, 2018).

**TABLE 1:** Conflicts according to the Operationalized Psychodynamic Diagnosis (OPD-2).

Conflict	Passive mode	Active mode
C1: Individuation vs. dependency	Existential fear of being left alone, high dependency on others	Excessive independency with a fear of closeness to others
C2: Submission vs. control	Submitting to others, e.g., tradition or other obligations	Striving for dominance and power to control situations
C3: Care vs. autarky	Attaching to others and demanding care	Not demanding anything from others, deferring own needs
C4: Self-worth conflict	Sense of shame and feeling worthless	Exaggerated self-confidence
C5: Guilt conflict	Feeling guilty, blaming oneself	Externalizing the feeling of guilt, blaming others
C6: Oedipal conflict	Restraint, submission, shyness and unremarkable appearance	Dramatic, sometimes erotic appearance, wanting to be noticed at all costs
C7: Identity conflict	Lack of identity	Exaggerated identity due to insecurity

#### 1.1.2. The level of structural integration

The level of structural integration axis conceptualizes personality functioning as the integration of psychological core functions regarding oneself and in relation to others (i.e., “objects” in psychoanalytic terminology) (OPD Task Force, 2008). The OPD-2 describes four structural domains (perception, regulation, communication, attachment), each of which consists of a self-related and an object-related dimension. The resulting eight structural dimensions are each assessed by three structural facets (see Table 2).

**TABLE 2:** Structural dimensions as defined in the level of structural integration axis in the Operationalized Psychodynamic Diagnosis (OPD-2).

Self	Objects
Self-perception	Object perception
● Self-reflection	● Self-object differentiation
● Affect differentiation	● Whole object perception
● Identity	● Realistic object perception
Self-regulation	Regulation of object relationships
● Impulse control	● Protecting relationships
● Affect tolerance	● Balancing of interests
● Self-worth regulation	● Anticipation
Internal communication	Communication with the external world
● Experience of affects	● Making contact
● Use of fantasies	● Affect communication
● Bodily self	● Empathy
Attachment to internal objects	Attachment to external objects
● Internalization	● Ability to form attachments
● Use of introjects	● Accepting help
● Variable and triangular attachments	● Detaching from relationships

#### 1.1.3. Relationship between conflicts and structural integration

In the psychodynamic tradition, unconscious conflicts and the degree of a patient’s personality or ego pathology (i.e., structural integration) have commonly been thought of as related (McWilliams, 2011). However, even with the operationalization of levels of structural integration and psychodynamic conflicts through the OPD, there have hitherto only been very few empirical studies on the relationship between them. Rudolf (2004) found the individuation vs. dependency conflict (C1) and the self-worth conflict (C4) to appear mostly at lower levels of structural integration (i.e., individuals with greater impairment in personality functioning), while the oedipal conflict (C6) and the submission vs. control conflict (C2) were more frequent at higher levels. In a study by Kaufhold et al. (2017) the individuation vs. dependency conflict (C1) was also significantly more frequent in lower levels of structural integration. Yet, compared to Rudolf (2004) the care vs. autarky conflict (C3) and the self-worth conflict (C4) emerged more frequently at higher levels of structural integration and no significant difference could be found for the submission vs. control conflict (C2). Due to the small sample of patients diagnosed with one of the other conflicts (i.e., C5–C7), Kaufhold et al. (2017) could not make any valid statement regarding the prevalence of these conflicts. In addition to these mixed findings, the implications of the studies are somewhat limited as they only show frequencies. A more frequent occurrence of the individuation vs. dependency conflict (C1) at lower levels of structural integration does not mean that this conflict can only occur at lower levels. Nor can the conclusion be drawn that a low level of structural integration is automatically associated with an individuation vs. dependency conflict (C1). More importantly, however, the studies have only assessed how conflicts and the general level of structural integration are related. Yet, a more fine-grained understanding of the relationship between the conflicts and the structural dimensions is needed to improve treatment planning. Further, it is an unanswered conceptual question whether the two axes are distinct or highly inter-related psychodynamic constructs.

### 1.2. Network analysis

A methodological approach with the potential to address these questions empirically is network analysis. Network analysis allows us to compute and visualize associations between several constructs. The unique advantage of this method is that all variables are considered simultaneously within one statistical model, allowing to estimate the relation between any two variables, while controlling for all other variables in the network (Borsboom and Cramer, 2013). In networks, all included variables are represented as *nodes* and their connections (e.g., partial correlations) are referred to as *edges*. In addition to the visualization of the statistical relations, centrality parameters can be used to quantify the inter-connectivity between the nodes and their relative importance within the network structure. The most central nodes are thought to be most influential, as they are highly connected with other nodes in the network (Borsboom et al., 2011; Borsboom, 2017). Moreover, *bridge nodes* can be identified, which are defined as the nodes that link two communities (Jones et al., 2019). Another topic of interest is the clustering (e.g., community detection) of the nodes in a network (Golino and Epskamp, 2017).

To the best of our knowledge, only one study so far has explored the association between OPD-2 constructs with the use of network analysis. Vierl et al. (2023) have explored how the OPD-2 constructs (i.e., interpersonal relations, active and passive modes of conflict coping, and level of structural integration) are related with each other and with psychopathology (i.e., depression and somatization). The investigated psychodynamic constructs were assessed by the Inventory of Interpersonal Problems (IIP-32; Thomas et al., 2011), the OPD conflict questionnaire (OPD-CQ; Benecke et al., 2018), and the short version of the OPD structure questionnaire (OPD-SQS; Ehrenthal et al., 2015). Depression and somatization were assessed with the Patient Health Questionnaire (PHQ; Kroenke et al., 2001, 2002). Vierl et al. (2023) used the global scores of each questionnaire as nodes, except for the OPD-CQ, where the active and passive modes of conflict coping were integrated as separate nodes into the network. They found that psychopathology and psychodynamic constructs formed separate clusters that were positively interrelated. The level of structural integration was found to play an important role in the network, as it was the most central node in the network and linked psychodynamic constructs to psychopathology. Regarding the associations between structural integration and conflicts, the level of structural integration was highly associated with the passive modes of conflict coping, while only small partial correlations were found with the active modes. This indicates that passive modes of conflict coping may more often be associated with lower levels of structural integration, while active modes may be more equally distributed across different structural levels. However, the authors have only used global scores as variables in the network, so that the association between specific conflicts with specific structural dimensions could not be analyzed. Further, the authors assessed the psychodynamic constructs with self-report questionnaires. Yet, since psychodynamic constructs are conceived as unconscious phenomena, expert interviews and observer ratings are considered the gold standard.

### 1.3. Aim of the present study

The aim of the present study was to explore the associations between psychodynamic conflicts and levels of structural integration according to the OPD-2 with the use of network analysis. The study is meant to overcome the methodological shortcomings in the study by Vierl et al. (2023), by using OPD-2 interview data and by including the conflicts and the structural dimensions as separate nodes into the network. The specific objectives of the study were (1) to examine the network structure to explore how conflicts and structural dimensions are inter-connected, (2) to investigate clusters in the network to determine whether the axes are statistically distinct constructs, (3) to identify the most central node(s) in the network and (4) to detect bridge nodes to examine which conflict is most strongly related to structural dimensions and vice versa.

Our objectives therefore were exploratory in nature, which fits well with network analysis which is commonly considered a tool for exploratory analyses. Nevertheless, in accordance with the assumptions made in the OPD, we expected the psychodynamic conflicts and structural dimensions to form distinct but interconnected clusters. Moreover, we expected particularly strong associations between the individuation vs. dependency conflict and structural dimensions, as this conflict has been previously found to be more often rated at lower levels of structural integration (Rudolf, 2004; Kaufhold et al., 2017).

## 2. Materials and methods

### 2.1. Participants

We investigated a sample of 228 adult outpatients, who were treated between 2012 and 2017 in one of five German clinical centers (Berlin, Hamburg, Heidelberg, Kassel, Munich). The study is a secondary analysis of data from the intake assessment prior to the experimental manipulation within a RCT study of patients with anxiety and personality disorders (Benecke et al., 2016). Use of this data for research purposes was approved by the ethics commission of the University Kassel (ethics vote of November 2nd, 2011). All patients gave their informed consent for the anonymous use of their data for scientific purposes.

Participants had a mean age of 37.8 years (*SD* = 11.6; range = 20–71 years) and 64.4% were female. Most of them (89.5%) had German citizenship. Fifty-three percent reported being married or in a stable relationship, 34.3% reported to not be in a relationship and 11.8% were divorced or widowed. Fifty percent had finished school with a higher education degree, 47.8% had a secondary school certificate and four individuals dropped out of secondary school. Almost half (48.5%) were currently employed, 17.8% were university students or in training, 20.3% were unemployed and 5.9% were retired.

According to Structured Clinical Interviews (SCID-I and II; Fydrich et al., 1997; Wittchen et al., 1997), all patients had at least one DMS-IV Axis 1 disorder (*M* = 2.9, *SD* = 1.53*;* range = 1–11). All met criteria for an anxiety disorder, 70.2% for an affective disorder, and 25.8% for a disorder from the somatoform spectrum. Moreover, 6.2% were diagnosed with a compulsive disorder, 4.9% with an eating disorder and 2.2% with a substance use disorder. In addition, all patients had at least one diagnosis of a PD according to DSM-IV criteria (American Psychiatric Association [APA], 1994). Almost forty percent (38.8%) were diagnosed with an avoidant PD, followed by compulsive PD (25.6%), depressive PD (18.5%), dependent PD (16.3%), Borderline PD (15.4%), and unspecified PD (13.7%). All other PDs were less frequent (<10%).

### 2.2. Measures

Semi-structured clinical OPD interviews were conducted and rated by OPD licensed and trained clinicians before the beginning of treatment. We used the OPD-2 axes conflicts (axis III) and levels of structural integration (axis IV).

As detailed above, the conflict axis captures seven psychodynamic conflicts. All conflicts were rated on a four-point Likert scale ranging from 0 (“absent“) to 3 (“very significant”). Further, the main conflict and the second most significant conflict were identified. Finally, the main conflict was rated as predominantly active, passive or a mixture of both modes. The conflicts were rated for all levels of structural integration, including low and disintegrated levels. At lower levels of structural integration, it is assumed that the conflicts are no longer stable or distinct dysfunctional patterns (i.e., “neurotic conflict”), but that the conflictual themes can become more diffuse and/or manifest themselves in an extreme way (e.g., existential fear of separation) (OPD Task Force, 2008). Such conflictual expressions were rated as “conflict schema.” Adequate inter-rater reliability of the OPD-2 has been shown before, with the ICC ranging between.52 and.64 for most conflicts, except for the identity conflict which showed insufficient inter-rater reliability (ICC = 0.08; Kaufhold et al., 2017). The identity conflict was excluded in this study because it did not occur frequently enough in our sample.

To assess the level of structural integration the OPD-2 offers a detailed operationalized checklist to rate the level of each structural dimension on a seven-point Likert-scale ranging from good (1), good—moderate (1.5), moderate (2), moderate—low (2.5), low (3), low—disintegrated (3.5) and disintegrated (4). Finally, the overall level of structural integration was rated. Adequate to good inter-rater reliability has been found before, with kappa values varying between 0.61 and 0.82 for the structural dimensions and 0.83 for the composite score (Benecke et al., 2009). Internal consistency for the overall level of structural integration has been reported to be α = 0.86 (Zimmermann et al., 2012).

### 2.3. Statistical approach

All statistical analyses were conducted with the statistical software *R*, v. 4.0.3 (R Core Team, 2020). The *R* code to reproduce the network analyses is available online,^1^ where we also provide the correlation and adjacency matrices to make the analyses reproducible.

#### 2.3.1. Descriptive analysis

We report the descriptive frequencies of the conflicts (i.e., rating of all conflicts, main conflict, second most important conflict), and the overall level of structural integration of the patients. Further, we aimed to determine the prevalence of specific conflicts in accordance to the overall level of structural integration. For this, we divided the group into patients with good and low levels of structural integration. Individuals with good, good—moderate, and moderate levels of structural integration (*n* = 153) were assigned to the first group. The patients with lower levels of structural integration formed the second group (*n* = 83). Group comparisons were calculated with the use of the Fisher’s exact test for categorical variables. The level of statistical significance was set as alpha <0.05.

#### 2.3.2. Network analysis

##### 2.3.2.1. Variable selection, missing data, and data transformation

For the network analysis we used the rating of all conflicts and of the eight structural dimensions. First, we inspected the item informativeness of all included constructs (see Supplementary Table 1). Importantly, the identity conflict showed an extremely skewed distribution (skewness = 3.42), with the conflict being absent or insignificant for 96.9% of the participants, while it had only been rated significant in one participant (<1%). Therefore, we removed the identity conflict from further analysis. In consequence, 14 variables were included in the network (i.e., six conflicts and eight structural dimensions).

Further, eight patients were excluded from the analysis, because more than a third of their values was missing. For the remaining 220 patients the missing values of the variables ranged between 0 and 15%. We imputed data ten times with the use of predictive mean matching as implemented in the *mice* package v. 3.14.0 (Van Buuren and Groothuis-Oudshoorn, 2011) and retained the mean value of the imputed datasets.

Lastly, the variables of the structural dimensions were measured in 0.5 steps which poses a problem for the network estimator used by *R*, since the variables were not recognized as ordinal. Therefore, all variables that were included in the network analysis were transformed by multiplying them by two, which turned the variables into integers that were correctly recognized by the network estimator.

##### 2.3.2.2. Network estimation

We estimated a regularized partial correlation network (i.e., Gaussian graphical model; GGM) using the *estimateNetwork* function from the *R* package *qgraph* v. 1.9.2 (Epskamp et al., 2022). The model with the best fit was selected via the Extended Bayesian Information Criterion (EBIC; Foygel and Drton, 2010) and the graphical least absolute shrinkage selection operator (glasso; Tibshirani, 1996), with the tuning parameter set to 0.5. This method is recommended for psychological networks with small sample sizes as it addresses the risk of false positive edges due to multiple testing, by shrinking spurious edges to zero and, therefore, only including edges in the network that likely represent true connections (Isvoranu and Epskamp, 2021). Since the variables were not normally distributed, we used spearman correlations for the network estimation (Epskamp and Fried, 2018). The network was computed and visualized using *qgraph* (Epskamp et al., 2022). In our network, edges (i.e., the links) between the nodes (i.e., variables) represent partial correlations, which are controlled for the influence of all other nodes in the network (Epskamp and Fried, 2018). Positive edge weights indicate that the connected nodes covary in the same direction (node A increases, node B increases), while negative nodes indicate that they covary inversely (node A increases, node B decreases) (Jones et al., 2019). The chosen layout for the network plot presents the two axes as two circles. This allows for an optical separation of conflicts and structural dimensions and improves a visual understanding of their interconnectedness.

##### 2.3.2.3. Clustering

To identify clusters in the network we used exploratory graph analysis (EGA; Golino and Epskamp, 2017) using the *EGAnet* package v.1.1.0 (Golino et al., 2022). EGA estimates a network followed by a multi-level modularity optimization algorithm to detect potential clusters. We applied the Louvain algorithm (Blondel et al., 2008), which has been shown to be better performing in continuous data than other algorithms (Christensen et al., 2021). The stability of the clusters was assessed with the use of 1,000 non-parametric bootstrap iterations using the *bootEGA* function.

##### 2.3.2.4. Network inference

To identify the most central nodes we calculated the strength centrality of the nodes using the centrality function in the *qgraph* (Epskamp et al., 2022). Strength centrality is defined as the sum of absolute edge weights that a node shares with all other nodes in the network (McNally, 2016). Thus, nodes with a high strength centrality are highly connected within the network. While other centrality measures exist, we decided to focus on strength centrality, as we wanted to have a measure of overall connectedness of the nodes in the network. As strength centrality may be influenced by differences in item variability (Terluin et al., 2016) we assessed spearman correlations between the strength centralities and the standard deviations of the items. If the correlation is significant, the nodes’ centrality may only be limitedly interpretable.

We additionally identified bridge nodes using the *bridge* function of the *R* package *networktools* v.1.5.0 (Jones, 2022). Bridge nodes are defined as the nodes that are linking two communities (here: psychodynamic conflicts and structural dimensions). We inspected bridge strength centrality, which reflects the sum of all absolute edge weights connecting a node from one community to all nodes from the other community (Jones et al., 2019).

Because both strength and bridge strength centrality are sample dependent, centrality difference tests were conducted via non-parametric bootstrapping (*nboots* = 2,500) using the *bootnet* package, v.1.5 (Epskamp et al., 2018). Centrality difference tests identify whether a given node’s (bridge) strength centrality is significantly greater than the (bridge) strength centrality of the other nodes within the network. Centrality indices should only be interpreted if there are significant differences between the nodes’ centralities (Levinson et al., 2018). We interpret nodes as the most central nodes that are more central than at least 50% of all other nodes in the network. Bridge nodes are required to show a greater bridge strength than at least 50% of the other nodes within the same community. Likewise, edge weight difference tests were conducted.

##### 2.3.2.5. Network stability

Network stability was assessed by bootstrapping 95% confidence intervals (*CI*) around edge weights (*nboots* = 2,500), and with correlation stability (*CS*) coefficients (*nboots* = 2,500), which were assessed for strength centrality, bridge strength centrality and edge weights. *CS*-coefficients over 0.5 imply strong stability (Epskamp and Fried, 2018).

## 3. Results

### 3.1. Descriptive analysis

The average level of structural integration in the sample was moderate (*M* = 2.18, *SD* = 0.4). Levels of structural integration were distributed as follows: good: *n* = 1 (0.4%), good—moderate: *n* = 17 (7.5%), moderate: *n* = 127 (57.7%), moderate—low: *n* = 52 (22.8%), low: *n* = 22 (1%), low– disintegrated: *n* = 1 (0.4%), disintegrated: *n* = 0 (0.0%). Considering the conflict ratings, the care vs. autarky conflict (C3) dominated in the sample (*M* = 2.14, *SD* = 0.91, range: 0–3), followed by the self-worth conflict (C4; *M* = 1.73, *SD* = 1, range: 0–3), and the individuation vs. dependency conflict (C1; *M* = 1.51, *SD* = 1.15, range: 0–4). In contrast, the identity conflict (C7) was least frequent (*M* = *0.15, SD* = 0.46, range: 0–3). The information on the main conflicts and second most significant conflicts was available for *n* = 197 individuals and is depicted in Table 3. Regarding the relationship between the main conflicts and the overall level of structural integration, the individuation vs. dependency conflict was significantly more often assigned at lower levels of structural integration (*p* < 0.001), while the need for care vs. autarky conflict dominated at higher levels of structural integration (*p* < 0.001). There was a tendency for the oedipal conflict to be rated more frequently at higher levels of structural integration (*p* = 0.05), yet only seven individuals were diagnosed with an oedipal conflict as the main conflict, preventing meaningful statements. The other conflicts were equally distributed across the levels of structural integration (see Table 4). As expected, at lower levels of structural integration, the conflicts were more often described as conflictual schemas. Specifically, the conflicts were described as schemas in more than two thirds (70.0%) of the patients with a low or low-disintegrated level of structural integration. Descriptive statistics for all structural dimensions and psychodynamic conflicts are displayed in the Supplementary Table 1.

**TABLE 3:** Frequencies of the conflicts as main conflict and second most significant conflicts.

	Main conflict; *N* (%)	2nd conflict; *N* (%)	Total; *N* (%)
Individuation vs. dependency	53 (26.9%)	18 (9.8%)	71 (18.6%)
Submission vs. control	11 (5.6%)	52 (28.3%)	63 (16.5%)
Care vs. autarky	93 (47.2%)	31 (16.8%)	124 (32.5%)
Self-worth conflict	31 (15.7%)	65 (35.3%)	96 (25.2%)
Guilt conflict	1 (0.5%)	7 (3.8%)	8 (2.1%)
Oedipal conflict	7 (3.6%)	11 (6.0%)	18 (4.7%)
Identity conflict	1 (0.5%)	0 (0%)	1 (0.3%)

The information of the main conflicts was missing for *n* = 31 and for the second most significant conflict for *n* = 45.

**TABLE 4:** Frequencies of the main conflicts in patients with higher levels and lower levels of structural integration.

	Higher levels *N* (%)	Lower levels *N* (%)	Fisher’s exact test
Individuation vs. dependency	21 (13.7%)	31 (37.3%)	<0.001
Submission vs. control	7 (4.6%)	3 (3.6%)	1.00
Care vs. autarky	78 (51.0%)	13 (15.7%)	<0.001
Self-worth conflict	19 (12.4%)	9 (10.85%)	0.83
Guilt conflict	1 (0.7%)	0	1.00
Oedipal conflict	7 (0.5%)	0	0.05
Identity conflict	0	1 (1.2%)	0.35

Patients with higher levels of structural integration were defined as individuals with good (1) to moderate (2) levels of structural integration (*N* = 153). Lower levels were defined as moderate—low integrated (2.5.) to disintegrated (4) levels of structural integration (*N* = 83).

### 3.2. Network estimation and stability

The network was found to be accurate and stable (edge CS-coefficient = 0.60, strength centrality CS-coefficient = 0.60, bridge strength CS-coefficient = 0.60), allowing reliable interpretations of the edge weights and the nodes’ (bridge) strength centralities (see Supplementary Figures 1–3). Moreover, bootstrapped CI of estimated edge-weights indicate accurate estimations of the edge weights (see Supplementary Figure 4).

**FIGURE 1:**
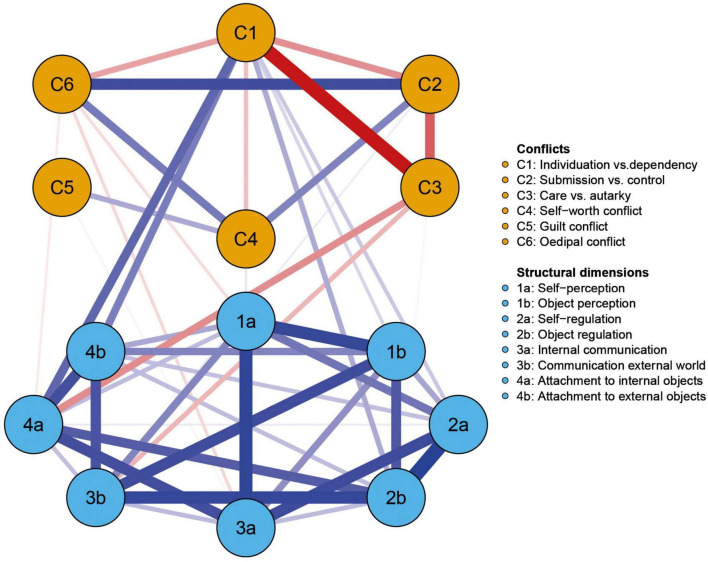
Visualization of the estimated network showing the partial correlations between psychodynamic conflicts (orange) and structural dimensions (blue). Red edges signify negative associations, blue edges positive ones. The brightness and thickness of the edge displays the strength of the association.

A visualization of the network and a description of the node labels is shown in Figure 1. Of the possible 91 edges 47 edges (51.6%) were evident in the network, with a mean edge weight of 0.038. An inspection of the edges within the network reveals that all eight structural dimensions were densely and positively connected with each other, with mostly no significant differences in their edge weights (see Supplementary Figure 5). Compared to the densely connected structural dimensions, the conflicts showed fewer and also negative edges. Interestingly, C1 showed exclusively negative associations to the remaining conflicts (except to C5, where no association emerged). C5 was solely associated with C4. Regarding the edges between the conflicts and structural dimensions, C1 was positively connected to several structural dimensions (i.e., 1b, 2a, 2b, 4a, 4b). In contrast, only negative edges emerged for C3 and for C6 with structural dimensions. Finally, only one weak edge emerged each for C2 and C5 with structural dimensions, while no association was found between C4 and any structural dimension. All edge weights can be found in the adjacency matrix online (see text footnote 1).

### 3.3. Clusters

The EGA community detection resulted in two distinct clusters for psychodynamic conflicts and structural dimensions. The clusters were found to be stable across 1,000 bootstrap iterations. For more details, please see the Supplementary Figures 8–10.

### 3.4. Network inference

**FIGURE 2:**
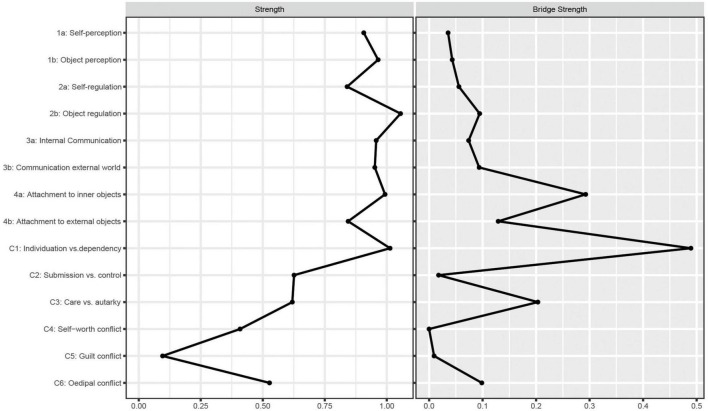
Strength centrality and bridge strength centrality of the nodes in the network.

Strength centrality (*S*) and bridge strength centrality (*BS*) indices are plotted in Figure 2, while the raw values can be found in the Supplementary Table 2. In our network no node was significantly more central than most (>50%) other nodes in the network. In detail, strength centrality was high for all structural dimensions (*S* ≥ 0.84), with no significant differences between them (see Supplementary Figure 6). Of the conflict axes, C1 showed the highest strength centrality (*S* = 1.01), which was significantly higher than the strength centrality of most other psychodynamic conflicts (except C2) but did not significantly differ from the strength centrality of any structural dimension (see Supplementary Figure 6). Strength centrality was not significantly correlated to standard deviations, suggesting that there is no potential relationship of variance to centrality.

Looking at the bridge strength centrality, C1 (*BS* = 0.49) and 4a (*BS* = 0.29) showed the highest BS values. Both are significantly higher than the bridge strength centrality of most other nodes in the network (see Supplementary Figure 7). The two nodes can therefore be considered the bridge nodes between the two axes in the network. The partial correlation between C1 and 4a was *r*_*p*_ = 0.15.

## 4. Discussion

This study is to our knowledge the first to examine the relationship between the individual psychodynamic conflicts and the separate structural dimensions according to the OPD using network analysis. The objectives of the current study were (1) to explore the general network structure (i.e., the edges) in the network, (2) to examine whether conflicts and structural dimensions form distinct clusters within the network, (3) to identify the most central nodes in the network, and (4) to detect bridge nodes. To address our research objectives, we analyzed OPD-2 interview data of *N* = 220 outpatients.

Overall, our network showed especially strong connections (i.e., edges) within the structural dimensions, while fewer and also negative edges were found within the psychodynamic conflicts. The individuation vs. dependency conflict (C1) showed several connections with structural dimensions, while other conflicts only showed few, or no associations with structural dimensions. Further, the results support the separation of psychodynamic conflicts and structural integration as distinct axes, as in the EGA community detection both were found to form separate clusters. Regarding strength centrality, C1 was found to be more central compared to most other conflicts in the network, while the strength centralities of the structural dimensions did not significantly differ from each other. Yet, no node was significantly more central to at least 50% of all other nodes within the network. Therefore, the statement that a specific node is most influential within the network is not admissible. Lastly, C1 and the capacity to attach to internal objects (4a) were identified as bridge symptoms. In the following, we highlight and discuss our findings in more detail.

Inspecting the network structure, it becomes apparent that the structural dimensions and the psychodynamic conflicts differ in their connectivity. The structural dimensions were strongly interconnected, supporting the idea that a total score of structure (i.e., global level of structural integration) can be meaningfully computed and interpreted when the individual structural dimensions are not of interest. The high connectivity between the dimensions also replicates previous research showing high inter-correlations between all structural dimensions (Doering et al., 2014). Regarding their centrality, we found no significant differences in the strength centrality of any structural dimension, indicating that no one dimension is particularly influential within the network.

In contrast, fewer and some negative connections were found between the conflicts. Remarkably, C1 showed exclusively negative edges to the other psychodynamic conflicts, with a particularly strong negative link to the care vs. autarky conflict (C3). The negative edges can be understood as follows: a profound C1 decreases the probability of the presence of any other conflict, but especially of C3. The strong negative association between C1 and C3 is particularly notable. This makes sense from our rating experiences with the OPD. Even though the manual does not rule out conceptually that both conflicts could both be very salient in one person, our clinical experience is that they are not. Both conflicts address dependency (or avoidance thereof) in relationships. However, they do so on different levels. C1 is about being dependent *on* a relationship, whereas C3 is about being dependent *within* a relationship. For patients with a strong C1 attachment and relationships (or their avoidance) are of existential importance, while for patients with a strong C3 it is less about the initiation of closeness or the avoidance of intimacy, but about the arrangement of the relationship in the sense of obtaining something from the other or providing for that other (OPD Task Force, 2008). In other words, patients with a strong C1 show more fundamental deficits in relationship formation, resulting in the question whether a close relationship (or lack thereof) can be tolerated at all–which seems to commonly make questions of care and being cared for (C3) of only secondary importance for the person. In order for a person to be very concerned with C3, it seems almost a prerequisite that the relationship as such is not the foremost issue.

This difference between the conflicts C1 and C3 is also highlighted by the associations of the conflicts with the structural ability to attach to inner objects (4a): C1 showed a strong positive link to 4a (i.e., a stronger manifestation of C1 is accompanied by more difficulties on this dimension), while a negative edge was found between 4a and C3 (i.e., a stronger manifestation of C3 is accompanied by fewer difficulties on this dimension). Attachment to internal objects contains the ability to develop and maintain emotional, stable internal images of significant others and to use these internal images for self-regulation. Moreover, it includes the ability to entertain variable and triadic relationships (OPD Task Force, 2008). Consequently, individuals with a strong C3 tend to have better abilities in these areas compared to patients with a strong C1. The difference found between the two conflicts and their association with attachment also fits with previous studies, were the attachment representation between OPD conflicts were compared (Müller, 1999). While C1 was frequently accompanied by insecure attachment representations, the representations were more secure for patients with a C3. This is also therapeutically of interest, in so far as that better abilities in attachment were associated with more positive outcome (Rudolf et al., 1996).

In the network, C3 also showed a negative association with the dimension “communication with the external world” (3b), which includes the ability to be in emotional contact with others, to communicate affects, and to be empathic. Again, the negative edge indicates that individuals with a strong C3 tend to have less impairments in this dimension. In contrast, positive associations were found between C1 and the structural dimensions “attachment to external objects” (4b), “self-regulation” (2a), “object regulation” (2b) and “object perception” (1b). Generally, the great number of positive edges with structural dimensions indicates a higher likelihood of many structural difficulties in patients with a C1. This fits to our findings that the C1 main conflict was significantly more often rated at lower levels of structural integration, while the C3 main conflict was significantly more often associated with better levels. This replicated the findings of Kaufhold et al. (2017). Our findings add to the literature by showing that these relationships result from associations with specific structural dimensions and mostly with the ability to attach to inner and external objects.

The other conflicts showed no or only very small associations with structural dimensions. The absence of an edge is supposed to represent conditional independence between two variables (Borsboom and Cramer, 2013), that is in this case: most conflicts were rather independent of the levels of structural integration, suggesting a distinctiveness of the axes. The separation of conflicts and structure as two distinct axes in the OPD is also supported by the EGA community detection analysis, which found that the psychodynamic conflicts and the structural dimensions formed two separate clusters in the network. Traditional psychoanalytic systems of nosology have often conflated conflict-based and structural aspects of personality pathology in their diagnostic categories (Christian, 2017). The data from the network analysis, however, supports the underlying assumption of OPD that conflict and structure, albeit interrelated, should be assessed separately. This perspective has been strengthened in the recently published third revision of OPD (OPD-3; OPD Task Force, 2023). In contrast to OPD-2, OPD-3 requires a rating of all conflicts regardless of the patients’ level of structural integration. The detailed assessment of psychodynamic conflicts is then not only standard-procedure for patients with a good level of structural integration (where the conflicts are referred to as “conflict tension”) and those with moderate levels of structural integration (“neurotic conflicts”) but also for patients with low levels of structural integration (“conflict schema”). This means that in the OPD-3 even for patients with severe impairments in their personality functioning, the dimension of unconscious motivational forces is addressed and can be taken into account to treatment planning and intervention. One example would be that conflicts could provide a better understanding of individual triggers and stressors that might be involved in the occurrence of destructive or self-destructive behavior. It is important to note here that even though conflicts and structural integration can be thought of as distinct axes, the expression of an unconscious conflict is supposed to differ depending on an individual’s level of structural integration. For example, the self-worth conflict shows an accentuated desire for recognition at a good level of structural integration, while a severe narcissistic personality disorder may be thought of at a lower-level expression of the same type of conflict. Yet, the conflictual motivational theme itself remains the same across all structural levels. In the case of C4 (self-worth), this is also reflected in the results of the network analysis: there is no edge between C4 and any structural dimension emerged, suggesting that the conflictual theme itself is independent of the level of structural integration. Also, C2 (submission vs. control) and C5 (guilt) only show a very small positive edge with structural dimensions each, so that, similarly to C4, the conflicts can occur at all levels of structural integration. Again, following the assumptions made in the OPD-3, the expression of the conflicts is supposed to differ across the structural levels, yet the conflictual motivational themes should remain the same. The oedipal conflict (C6) shows some (small) negative edges with structural dimensions (i.e., 1a, 3a, 4a). This indicates that the conflict occurs somewhat more often at better structural levels, which corresponds to the findings of Rudolf (2004). However, the small edges also show that the differences in frequency do not seem to be particularly large. The difference between C1 and C3 has already been described above: while C1 shows exclusively positive edges with structural dimensions, C3 shows only negative associations. Consequently, C1 is supposed to appear more frequently at lower levels of structural integration, while C3 is more frequent at better structural levels.

Lastly, C1 and the ability to attach to inner objects (4a) were identified as bridge nodes. From a network perspective targeting bridge nodes through interventions could have especially large impacts, as they may have therapeutic effects in both clusters (McNally, 2016). Our findings suggest that for patients with a strong C1 a conflict-focused treatment may also improve the levels of structural integration. Likewise, by targeting structural impairments the dynamic between individuation and dependency may also soften, particularly when focusing on attachment. For the other psychodynamic conflicts, the network analysis showed low bridge centrality which suggests that a change in conflict pathology is less likely to occur as a by-product of improving structural abilities. This has important clinical implications, including that psychotherapy and treatment planning for patients with lower levels of structural integration will likely also benefit from a thorough assessment of psychodynamic conflicts.

### 4.1. Strengths, limitations, and future research

The present study extends current knowledge on hybrid-dimensional-categorical diagnosis by exploring the associations between conflicts and structural dimensions according to the OPD. A particular strength of this study as opposed to previous work is the usage of OPD-2 interview data. Since psychodynamic constructs are thought to be at least partly unconscious, self-report questionnaires may not be ideal for capturing the constructs properly. Additionally, the interview data enabled us to do a more fine-grained analysis of the levels of structural integration through also considering the individual subscales (structural dimensions) instead of only relying on the total score. This allows to detect association patterns between the conflicts and different aspects of structural integration, which had not been possible in a previous study (Vierl et al., 2023).

Despite these strengths, several limitations must be mentioned as well. First, the sample size was rather small. However, the stability parameters found good stability, allowing us to draw reliable interpretations. Moreover, the analyzed data is cross-sectional, which prohibits to draw causal interpretations of the results. For example, the causal relationship between the individuation vs. dependency conflict (C1) and the structural dimension remains unclear. According to psychodynamic theory one would suggest that the fundamental deficits in relationship formation that are shown in patients with a strong C1 are more likely to be the consequence of impaired abilities in e.g., attachment and self/object regulation than vice versa. Intensive longitudinal data that includes how significant impairments in an individual’s life affect the network structure would be necessary to draw stronger conclusions. Additionally, the network is based on group-level data and cannot be applied directly to an individual. Individualized networks derived from time-series data would be needed to allow for personalized clinical recommendations (Bringmann, 2021). Moreover, our sample consisted of patients who were all diagnosed with at least one Axis I disorder (with all showing an anxiety disorder) and with at least one PD according to the DSM-IV. Consequently, the sample represents a rather impaired clinical sample. Further, only outpatients were considered. It remains unclear whether the findings can be generalized to other clinical or non-clinical samples. Another limitation concerns the assessment of psychodynamic conflicts: we were not able to take the different modes of conflict coping (passive vs. active) into account, since in the OPD-2 interview this information is only rated for the main conflict. This limits the conclusions we can draw from the network, as the study by Vierl et al. (2023) highlights the overall importance to differentiate between the modes of conflict coping. Moreover, the guilt conflict and oedipal conflict were less frequent in our sample (see Supplementary Table 1), which could have affected the results. Further, in 70% of all patients with low or low-disintegrated levels of structural integration, the conflicts were described as conflict schema. Due to the small sample, we could not compare the associations between “neurotic conflicts” and “conflict schema.” In OPD-3, it is assumed that conflict schemas differ from neurotic conflicts in that the conflictual themes are more diffuse or show themselves in a more extreme way. Therefore, when comparing the networks for patients with neurotic conflicts and conflict schema, it could be that differences in the associations emerge. This could be the focus for future research. Lastly, we did not include the OPD axis concerning interpersonal relations. In the OPD-2 only the three most important relation patterns are rated per individual. While this is useful from a therapeutic point of view, a scientific analysis of the data is difficult because the data are highly incomplete.

## 5. Conclusion

In the new conceptualizations of PDs in the DSM-5 AMPD and the PD chapter in the ICD-11 PDs are assessed along a continuum of personality functioning and are further described with the help of personality traits. However, the relationship between personality functioning and personality traits has been controversially debated. An empirically founded understanding of this relationship is relevant for the parsimony of the diagnostic system on the one hand and for how it may inform treatment planning and case conceptualization on the other hand. Variants of these new conceptualizations had already been in clinical use as part of certain psychodynamic systems, such as the OPD. Yet, empirical research on the inter-relationship between psychodynamic constructs has still been missing. Therefore, in the present study we explored the inter-relationship between structural integration and psychodynamic conflicts according to the OPD. For this, we used OPD-2 interview data of *N* = 220 outpatients and conducted a network analysis. Our results showed that psychodynamic conflicts and the structural dimensions indeed form separate but connected clusters, supporting the conceptualization of conflicts and the level of structural integration as distinct axes in the OPD. The individuation vs. dependency conflict (C1) showed only negative edges with other conflicts, with a particularly strong negative association with the care vs. autarky conflict (C3). Moreover, C1 was strongly related to several structural dimensions, while most other conflicts showed only few or no connections to the structural dimensions. This shows that most psychodynamic conflicts are rather independent of the structural abilities. Thus, the conflicts can theoretically appear at all structural levels, even if they differ in frequency at the overall level of structural integration. At lower levels of structural integration, the conflicts were mostly described as “conflict schema.” The OPD-3 describes differences in conflict expression for “neurotic conflicts” and “conflict schema” while the underlying conflictual theme remains the same. Nonetheless, conflict schemas can contain important diagnostic information. A profound diagnosis of all conflicts is therefore recommended for all patients.

## Data availability statement

The data analyzed in this study is subject to the following licenses/restrictions: The analytic code for all analyses performed in this study is available along with the correlation and adjacency matrices online at https://osf.io/pkh9t/. These matrices can be used to assess our analyses. Requests to access these datasets should be directed to CB, benecke@uni-kassel.de.

## Ethics statement

The studies involving human participants were reviewed and approved by Ethics Commission of the University Kassel. The patients/participants provided their written informed consent to participate in this study.

## Author contributions

CV and LV performed statistical analysis and wrote the first draft of the manuscript. YH supervised the analytical methods. LV, CV, YH, CB, and CS discussed the results. LV, YH, CB, and CS revised the manuscript. All authors contributed to the article and approved the submitted version.

## Footnotes

1
https://osf.io/pkh9t/
