# Supplementary material for: How are psychodynamic conflicts associated with personality functioning? A network analysis
Source: Front Psychol. 2023 Apr 19;14:1152150. doi: 10.3389/fpsyg.2023.1152150 (PMC10155610; doi:10.3389/fpsyg.2023.1152150)
Supplement: Supplementary file 1 [file Table_1.pdf]

Supplementary Materials for:

How are Psychodynamic Conflicts Associated with Personality Functioning? A Network Analysis

Vierl, L.*, Von Bremen, C., Hagmayer, Y., Benecke, C., Sell, C.

* Corresponding Author: larissa@vierl.net

Supplementary Tables and Figures

Table S1: Item characteristics

Table S2: Raw values of strength centrality and bridge strength centrality

Figure S1: Edge weight correlation stability

Figure S2: Strength centrality correlation stability

Figure S3: Bridge strength centrality correlation stability

Figure S4: Edge weight bootstrap

Figure S5: Edge weight difference test

Figure S6: Centrality difference test

Figure S7: Bridge centrality difference test

Figure S8: Exploratory graph analysis (EGA)

Figure S9: bootEGA network plot

Figure S10: EGA item stability

Table S1

Item characteristics

Construct	Label	M (SD)	Skewness	Kurtosis
**Psychodynamic conflicts**				
Individuation vs. dependency	C1	1.51 (1.15)	0.06	1.64
Submission vs. control	C2	1.22 (0.95)	0.29	2.40
Care vs. autarky	C3	2.14 (0.91)	-0.71	2.48
Self-worth conflict	C4	1.73 (1.0)	-0.27	2.01
Guilt conflict	C5	0.69 (0.75)	0.98	4.10
Oedipal conflict	C6	0.72 (0.90)	1.02	3.02
Identity conflict	C7	0.15 (0.46)	3.42	15.34
**Structural dimensions**				
Self-perception	1a	2.06 (0.42)	0.45	3.49
Object perception	1b	2.16 (0.48)	0.07	2.49
Self-regulation	2a	2.22 (0.38)	0.89	3.25
Object regulation	2b	2.17 (0.44)	0.29	2.79
Internal communication	3a	2.17 (0.43)	0.08	3.21
Communication external world	3b	2.04 (0.46)	0.41	2.81
Attachment to inner objects	4a	2.18 (0.40)	0.53	3.65
Attachment to external objects	4b	2.06 (0.38)	0.76	4.90

Table S2

Raw values of strength centrality and bridge strength centrality

Construct	Label	Strength centrality	Bridge strength centrality
**Psychodynamic conflicts**			
Individuation vs. dependency	C1	1.01	0.49
Submission vs. control	C2	0.63	0.02
Care vs. autarky	C3	0.62	0.20
Self-worth conflict	C4	0.41	0.00
Guilt conflict	C5	0.10	0.01
Oedipal conflict	C6	0.53	0.10
**Structural dimensions**			
Self-perception	1a	0.91	0.04
Object perception	1b	0.96	0.04
Self-regulation	2a	0.84	0.06
Object regulation	2b	1.06	0.09
Internal communication	3a	0.96	0.07
Communication external world	3b	0.95	0.09
Attachment to inner objects	4a	0.99	0.29
Attachment to external objects	4b	0.84	0.13

Figure S1

Edge weight correlation stability

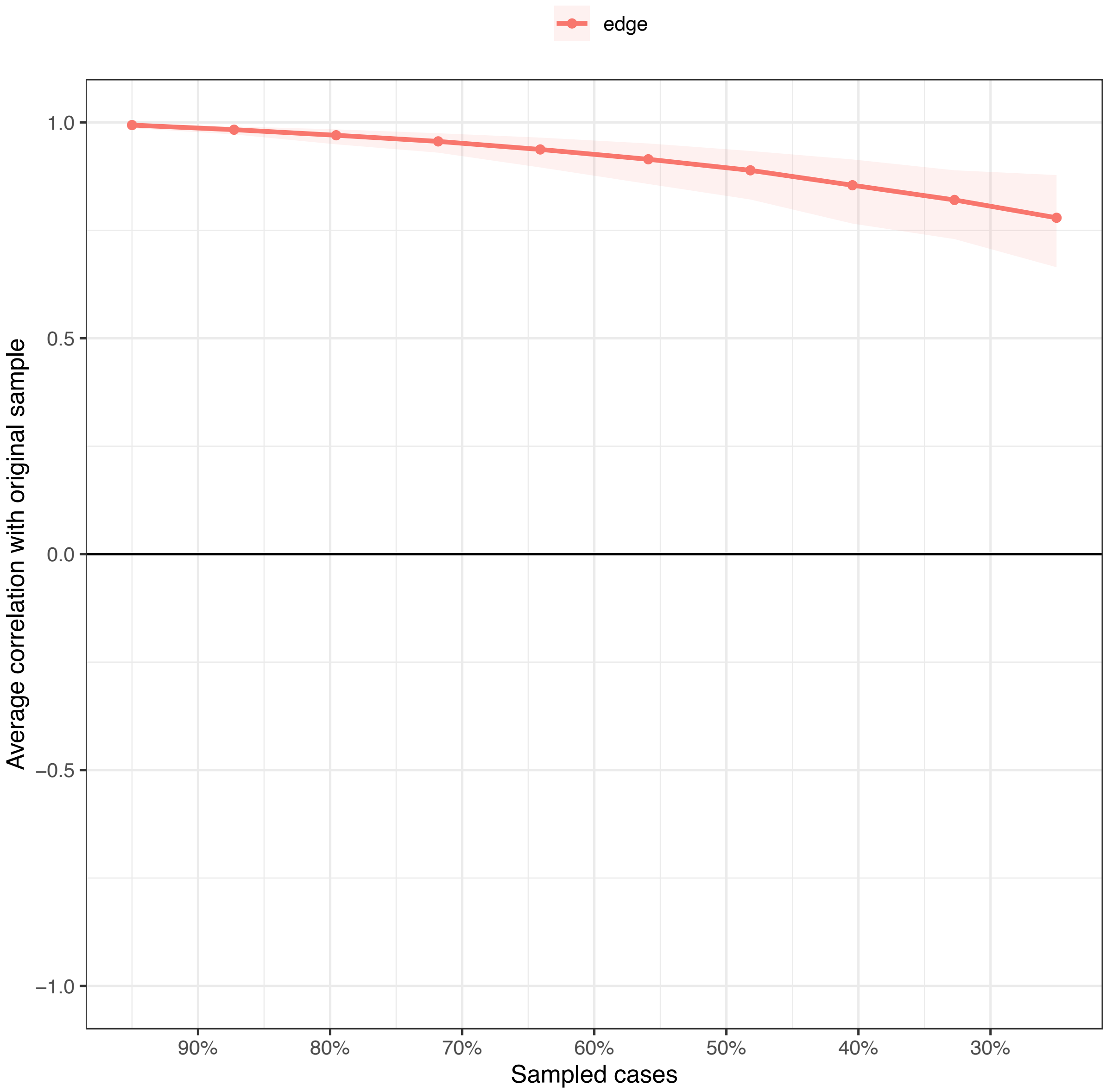

Note. The average correlation between the original edge weights and the edge weights after dropping a percentage of subjects at random from the data. The line represents how the edge weights change when dropping different proportions of the data. The straighter the line, the more reliable the edge weights. In our network, the plot and the corresponding edge correlation stability (CS)-coefficient of .595 indicate very stable and reliable edge weights.

Figure S2

Strength centrality correlation stability

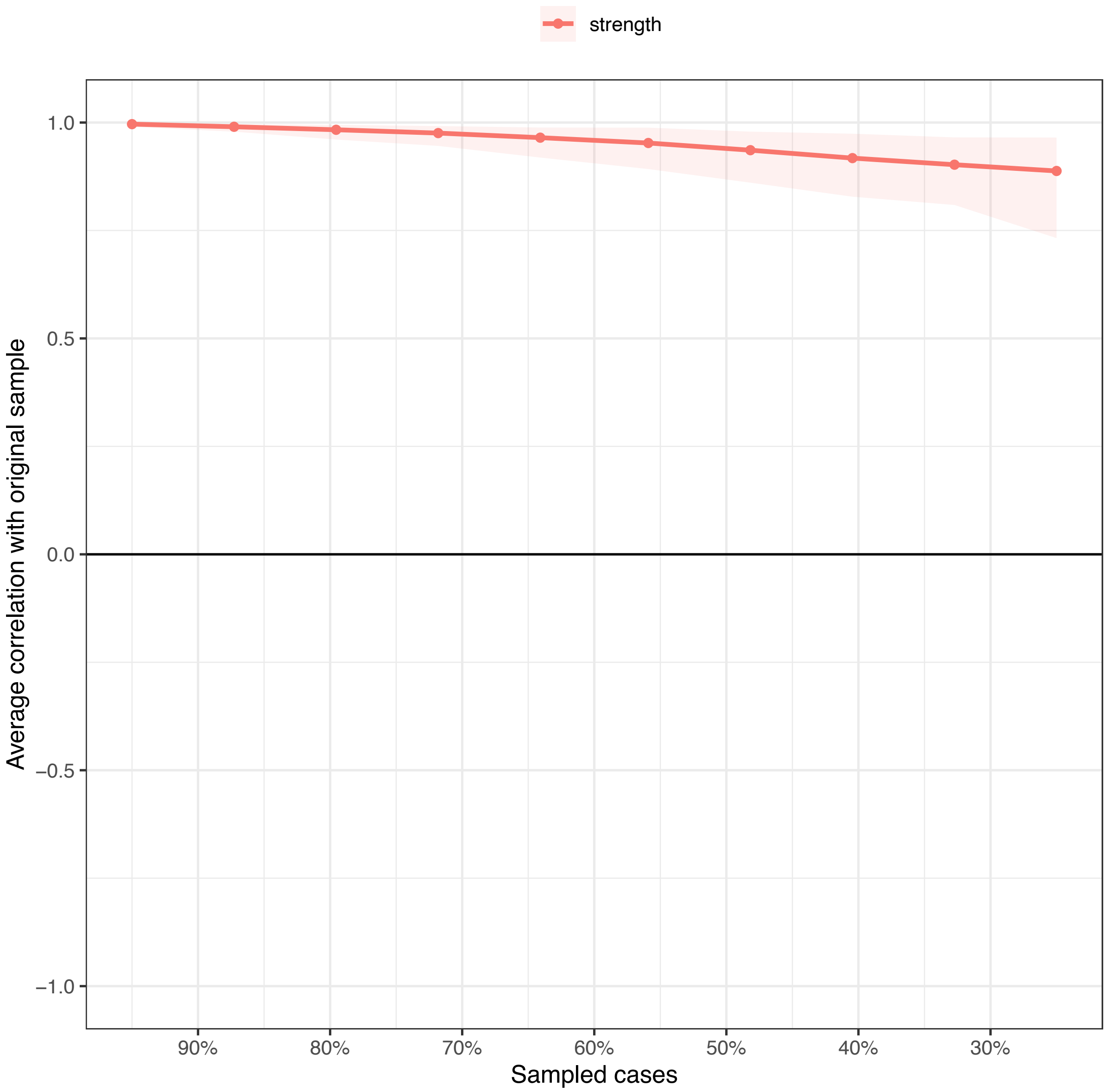

Note. The average correlation between the original strength centrality index and the strength centrality index after dropping a percentage of subjects at random from the data. The line represents how the strength centrality of the nodes changes when dropping different proportions of the data. The straighter the line, the more reliable the centrality. In our network, the plot and the corresponding strength centrality correlation stability (CS)-coefficient of .595 indicate very stable and reliable strength centralities.

Figure S3

Bridge strength centrality correlation stability

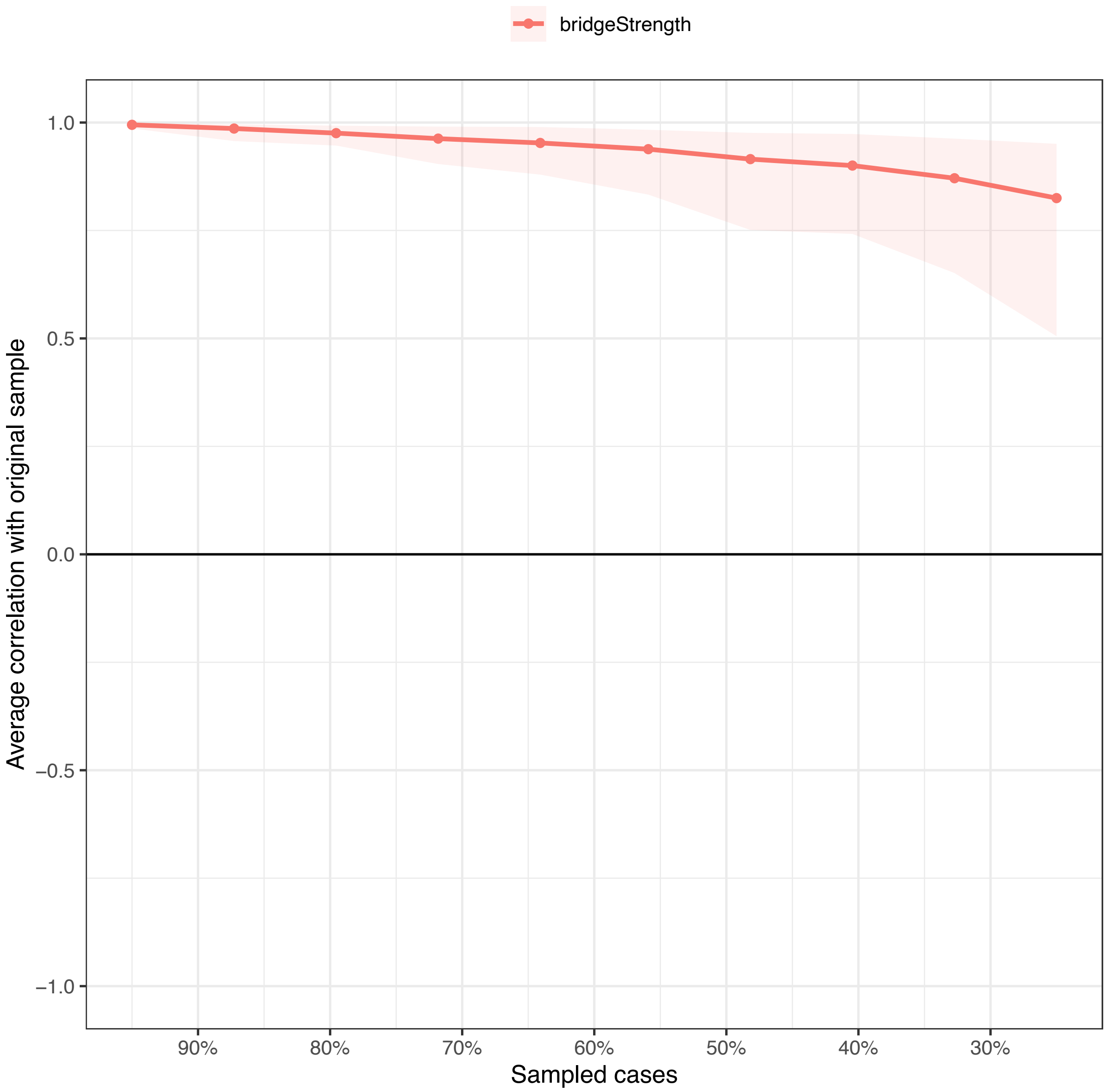

Note. The average correlation between the original bridge strength centrality index and the bridge strength centrality index after dropping a percentage of subjects at random from the data. The line represents how the bridge strength centrality of the nodes changes when dropping different proportions of the data. The straighter the line, the more reliable the centrality. In our network, the plot and the corresponding bridge strength centrality correlation stability (CS)-coefficient of .595 indicate very stable and reliable bridge strength centralities.

Figure S4

Edge weight bootstrap

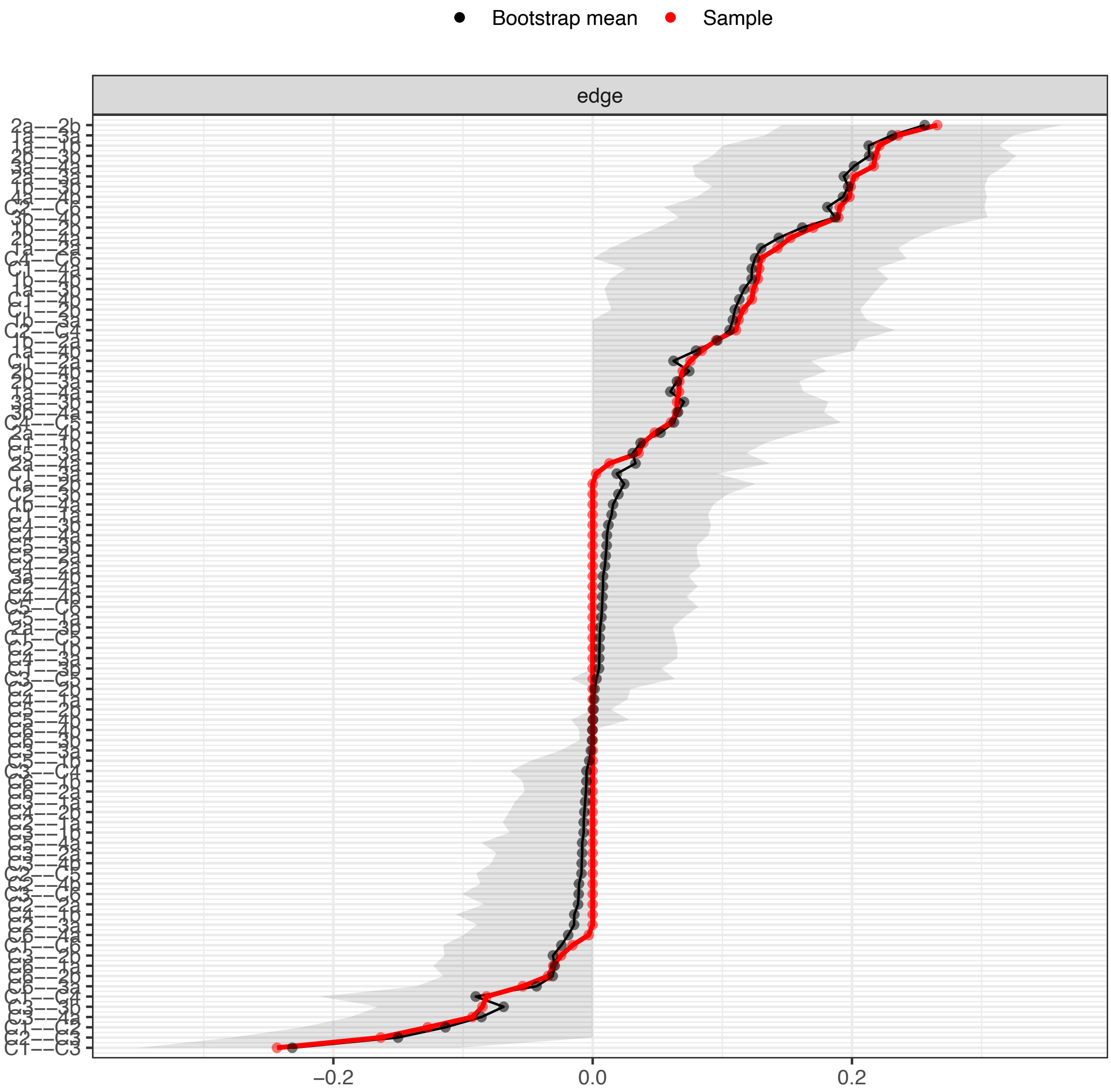

Note. Bootstrapped confidence intervals (*CI*s) of the estimated edge weights in the network across *n* = 2500 bootstraps. The red line indicates the original edge weight values, the black line the bootstrap mean edge weight values and the gray-shaded area the bootstrapped 95% *CI*s of the edge weight values. The sample values lie within the bootstrapped CIs and the bootstrapped mean edge values are relatively close to the edge weights in the original network, thus indicating accurate estimations.

Figure S5

Edge weight difference test

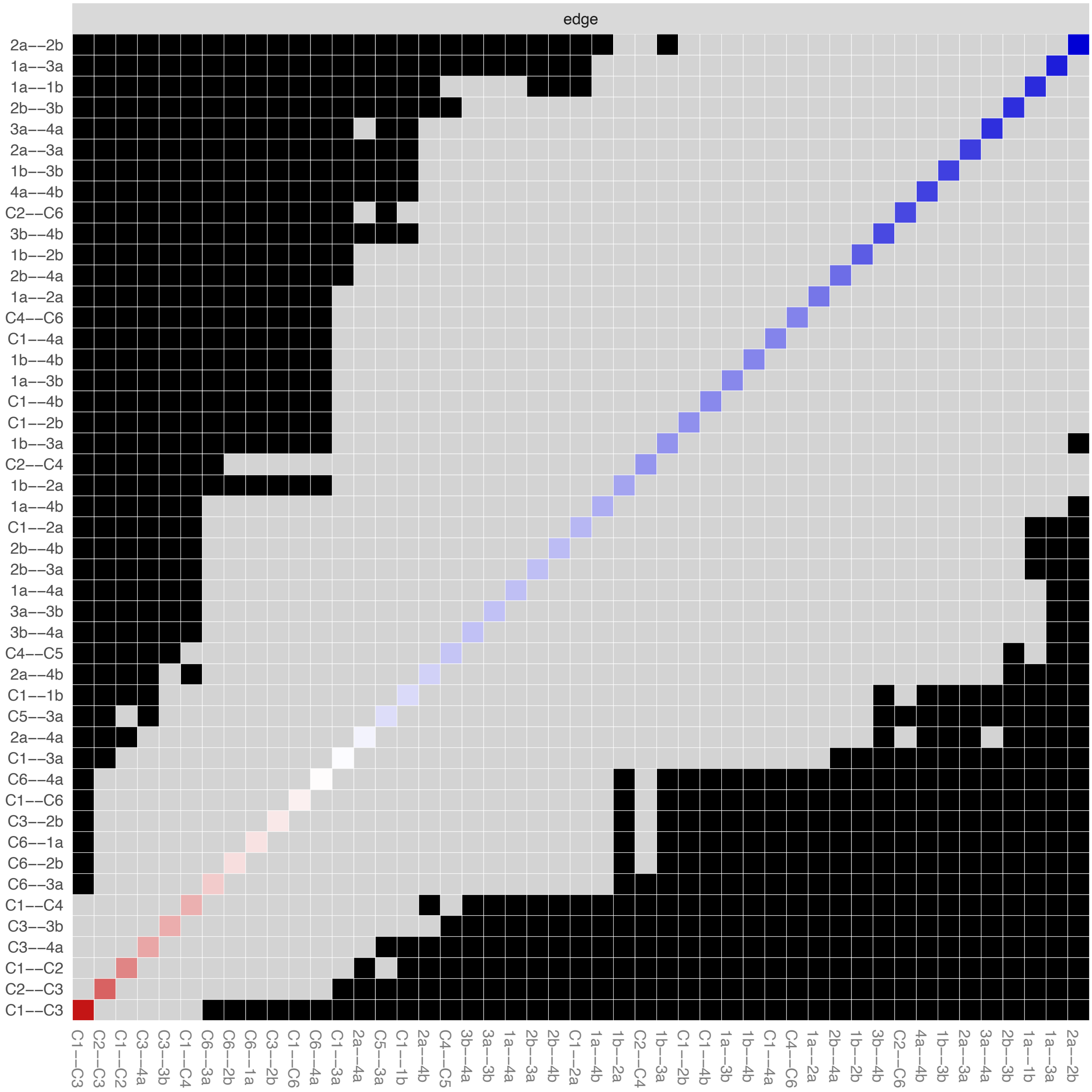

Note. The plot shows the differences between all pairs of edge weights (*nboots* = 2500). Each row and column indicate an edge weight. Black boxes represent significant differences between edge weights (α = 0.05), whereas gray boxes indicate non-significant differences. The color in the diagonal corresponds with the edge colors in the original network figure.

Figure S6

Strength centrality difference test

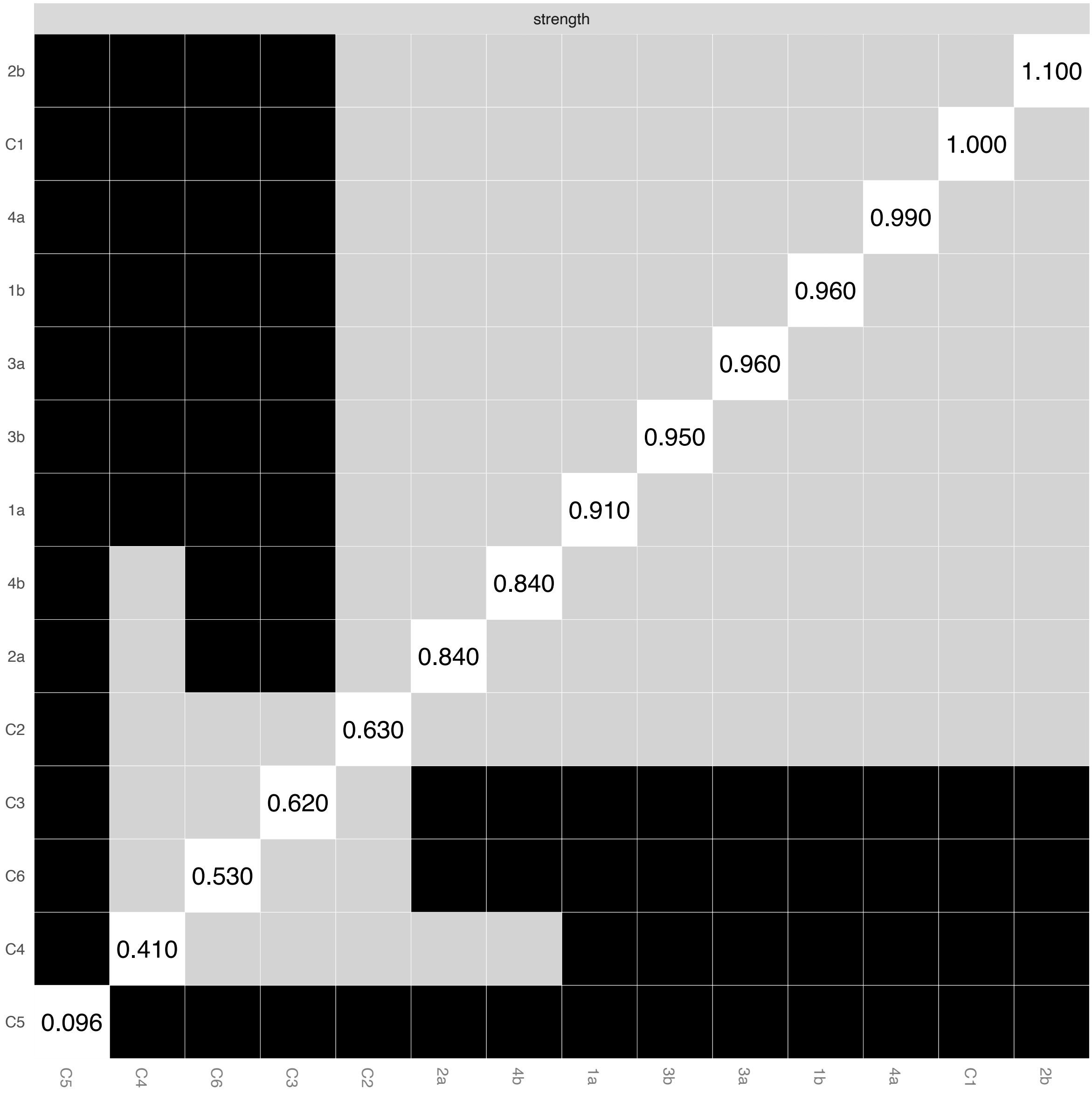

Note. The plot shows the differences between the strength centrality between all nodes (*nboots* = 2500). Each row and column indicate a node. Black boxes represent nodes that do differ significantly from one-another in their strength centrality (α = 0.05), gray boxes indicate non-significant differences. The values in the white boxes correspond with the value of the node's strength centrality in the original network figure.

Figure S7

Bridge strength centrality difference test

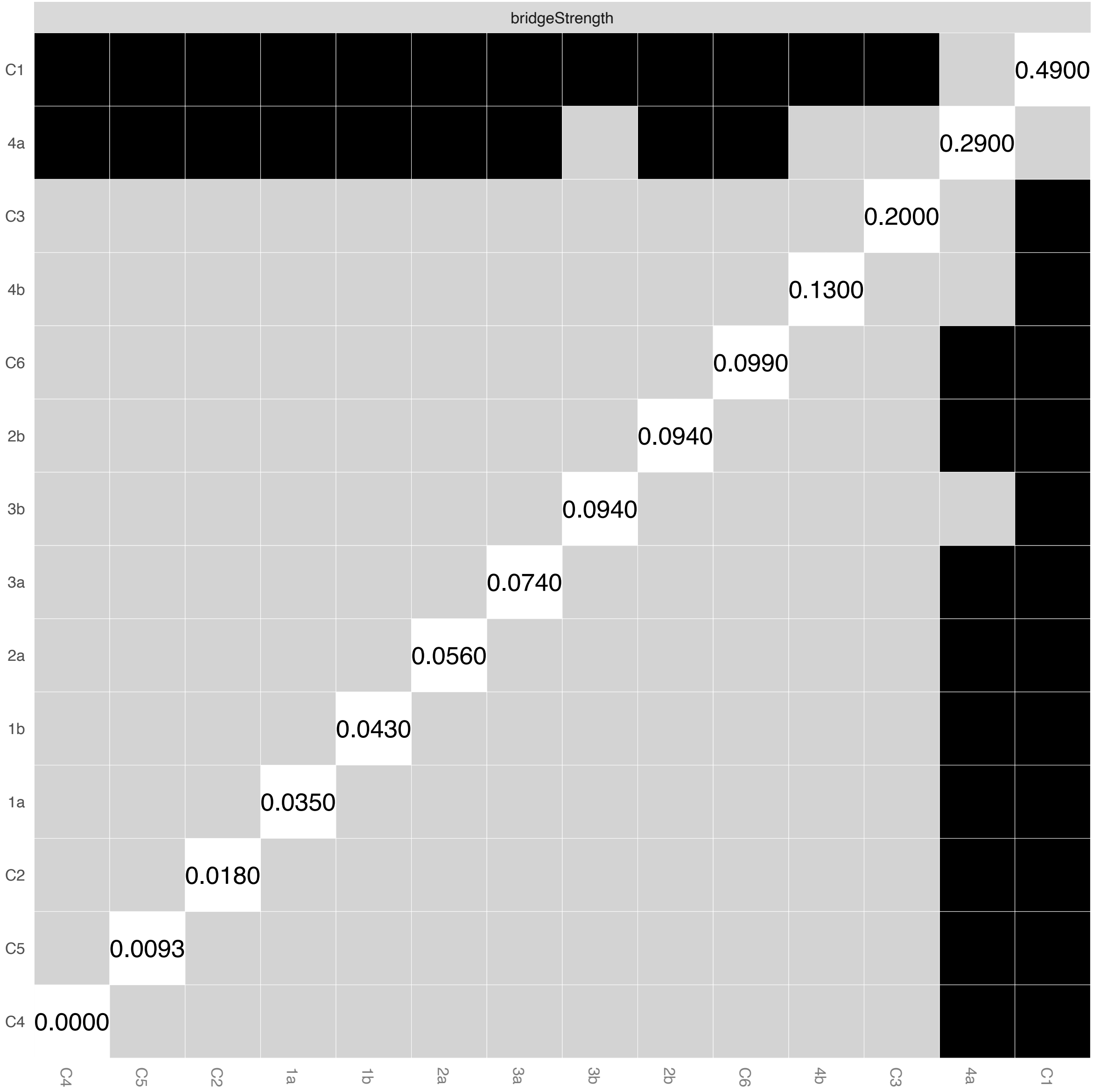

Note. The plot shows the differences between the bridge strength centrality between all nodes (*nboots* = 2500). Each row and column indicate a node. Black boxes represent nodes that do differ significantly from one-another in their bridge strength centrality (α = 0.05), gray boxes indicate non-significant differences. The values in the white boxes correspond with the value of the node's bridge strength in the original network figure.

Figure S8

Exploratory graph analysis (EGA)

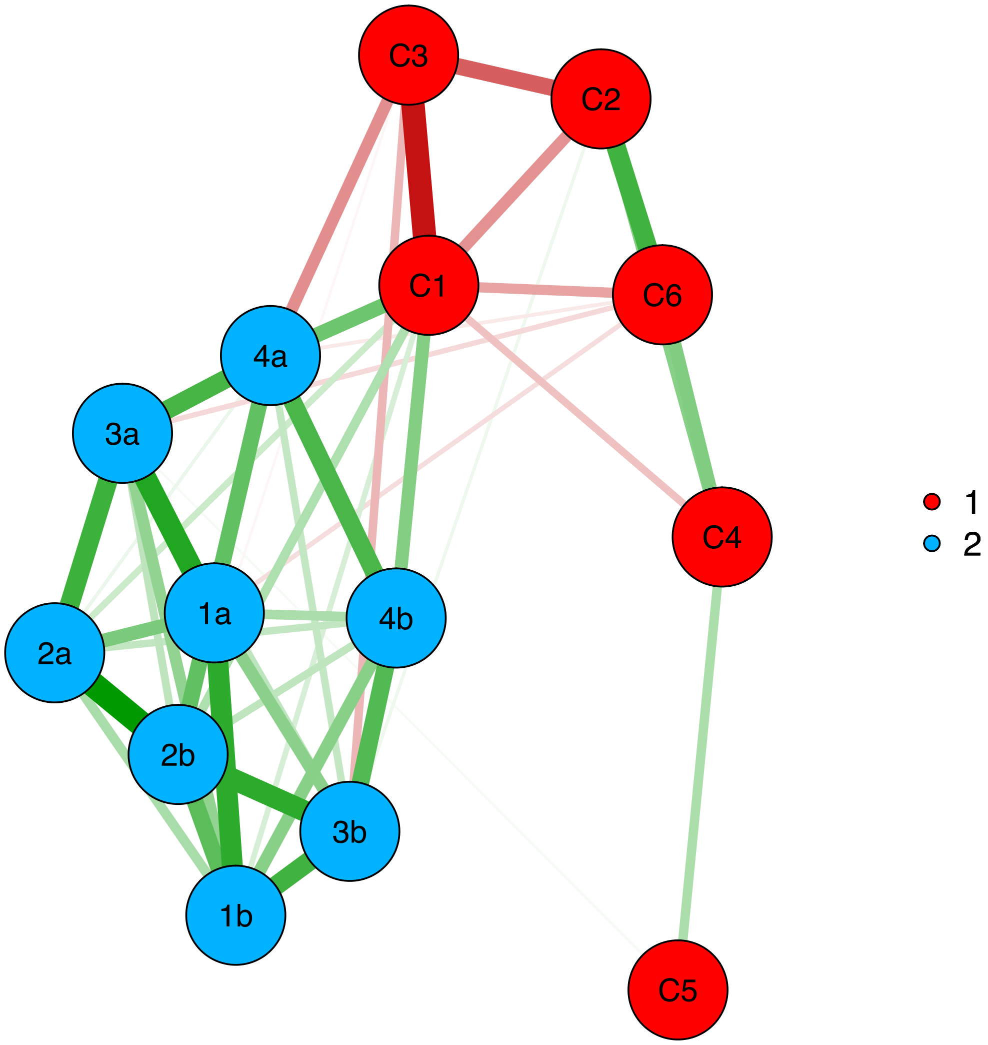

Note. Network model visualizing the clusters detected using exploratory graph analysis (EGA). The network layout used a modified version of the Fruchterman-Reinhold algorithm, placing more connected nodes closer to one another. The colors of the nodes represent the clusters resulting from the EGA, with blue nodes representing structural dimensions and red nodes representing psychodynamic conflicts. Green edges represent positive associations, red edges represent negative associations. Thicker edges represent stronger associations. See Supplementary Table S1 for item descriptions.

Figure S9

BootEGA network plot

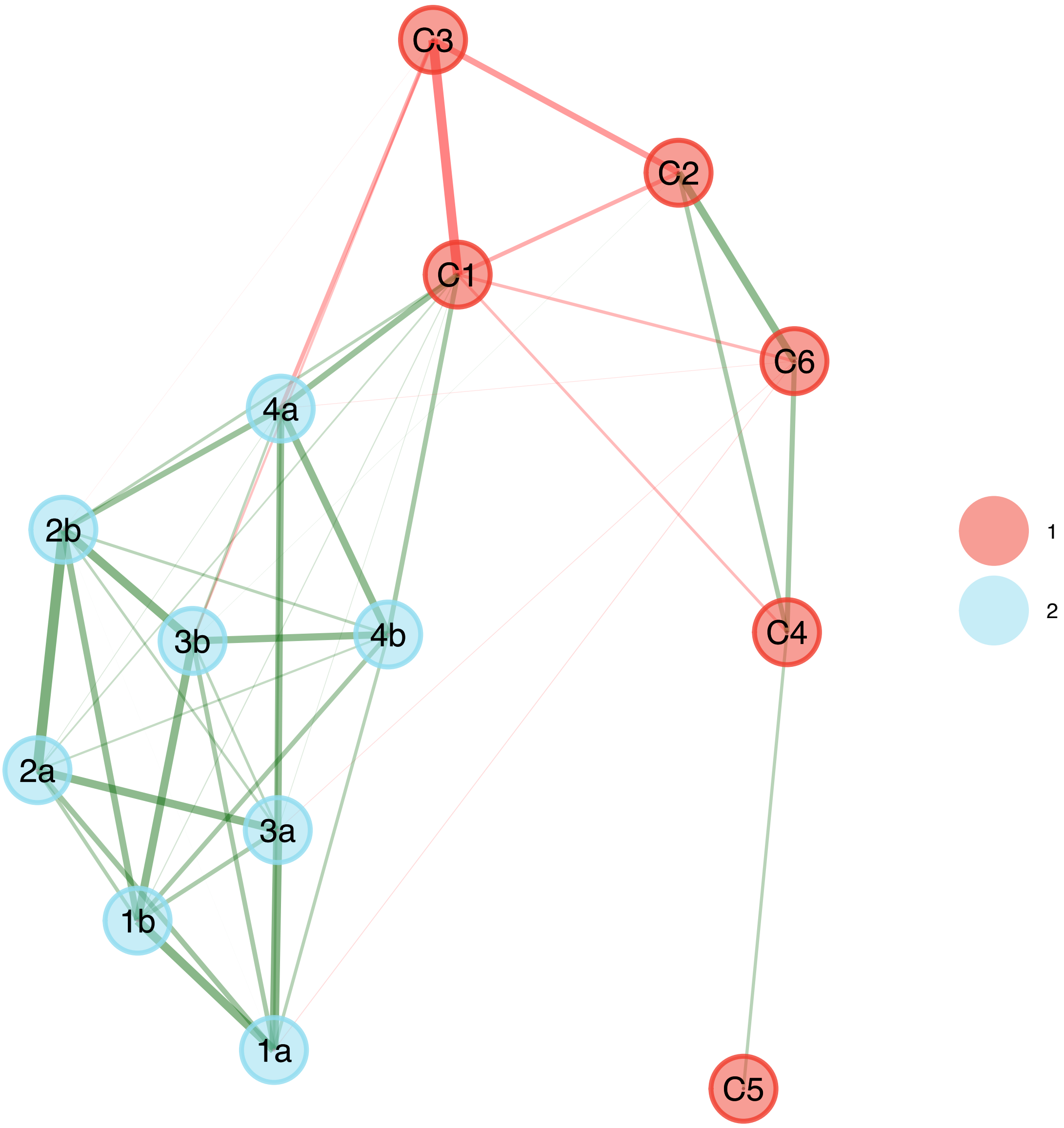

Note: The network plot visualizes the most typical EGA network structure across *n* = 1000 bootstrap iterations. The network replicated the original EGA clusters, therefore supporting their reliability.

Figure S10

EGA item stability

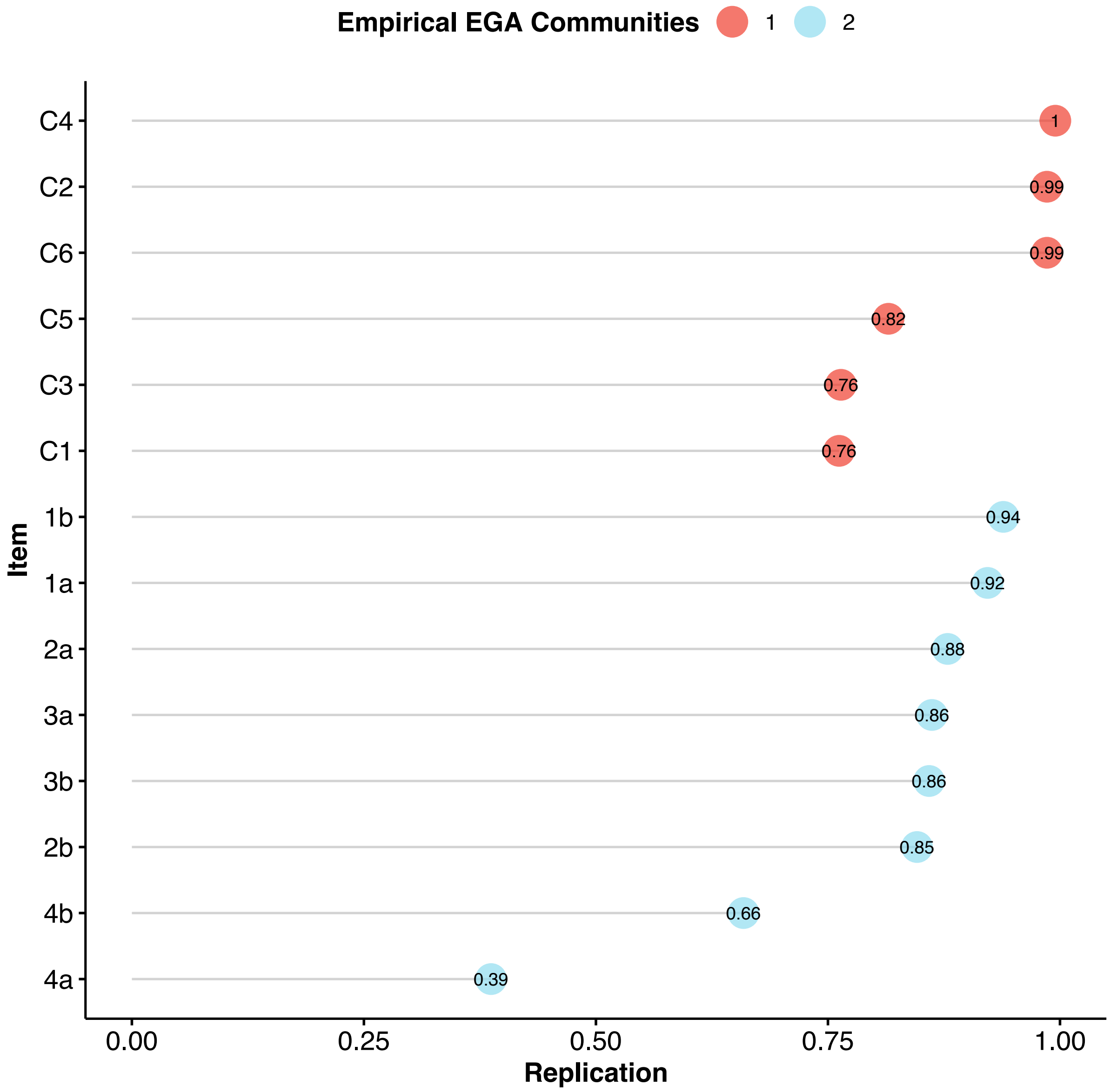

Note: The plot visualizes the item stability. The numbers in the nodes represent the proportion of times an item is replicated in the cluster specified by EGA across n = 1000 iterations during bootstrap analysis.
